# Pattern contrast influences wariness in naïve predators towards aposematic patterns

**DOI:** 10.1038/s41598-020-65754-y

**Published:** 2020-06-08

**Authors:** C. G. Halpin, O. Penacchio, P. G. Lovell, I. C. Cuthill, J. M. Harris, J. Skelhorn, C. Rowe

**Affiliations:** 1 Centre for Behaviour and Evolution, Newcastle University Biosciences Institute, Newcastle University, Henry Wellcome Building, Framlington Place, Newcastle upon Tyne, NE2 4HH UK; 2 School of Psychology and Neuroscience, South Street, University of St Andrews, St Andrews, Fife, KY16 9JP United Kingdom; 3 Division of Psychology and Forensic Sciences, School of Applied Sciences, Abertay University, Dundee, DD1 1HG United Kingdom; 4 School of Biological Sciences, University of Bristol, Bristol Life Sciences Building, 24 Tyndall Avenue, Bristol, BS8 1TQ United Kingdom

**Keywords:** Animal behaviour, Behavioural ecology

## Abstract

An apparent and common feature of aposematic patterns is that they contain a high level of achromatic (luminance) contrast, for example, many warning signals combine black spots and stripes with a lighter colour such as yellow. However, the potential importance of achromatic contrast, as distinct from colour contrast, in reducing predation has been largely overlooked. Here, using domestic chicks as a model predator, we manipulated the degree of achromatic contrast in warning patterns to test if high luminance contrast in aposematic signals is important for deterring naïve predators. We found that the chicks were less likely to approach and eat prey with high contrast compared to low contrast patterns. These findings suggest that aposematic prey patterns with a high luminance contrast can benefit from increased survival through eliciting unlearned biases in naïve avian predators. Our work also highlights the importance of considering luminance contrast in future work investigating why aposematic patterns take the particular forms that they do.

## Introduction

Aposematic prey advertise their defences (such as toxic chemicals, spines and irritating hairs) to potential predators using distinctive conspicuous colours and patterns^1–3^. Classic examples of aposematic coloration include the characteristic banded pattern of coral snakes, the yellow and black stripes of wasps and the spotted patterns of ladybirds^2,4^. Aposematic prey benefit from their warning signals because they can elicit unlearned aversions in naïve predators and accelerate the speed with which predators learn to avoid them in future encounters^2,5^. Indeed, there is now a considerable body of work showing that warning signals, or particular attributes of these colour patterns (e.g. hue, conspicuousness against the background, and the spatial arrangement of pattern elements) are particularly effective at enhancing predator avoidance^6–17^. However, the effect of one key aspect of warning signals has yet to be investigated directly: the degree of achromatic contrast in the patterns.

This is a crucial oversight since patterns with high internal luminance contrast (i.e. those with many borders between light and dark pattern elements) are almost a defining feature of an aposematic signal^18^, and there are a number of reasons to believe that such patterns are particularly effective. For example, avian predators may find patterns with high levels of luminance contrast intrinsically more visually stimulating than uniform coloration^19^: luminance contrast is used in both edge detection and texture discrimination^20,21^, which is important for object recognition^22,23^. This may make patterns with high luminance contrast easier for birds, and other predators, to recognise and remember^24^ and could help to facilitate colour avoidance learning^25^.

In addition, predators do appear to pay attention to luminance contrast when making foraging decisions^26,27^, and there is some evidence that luminance contrast in aposematic signals may be important in deterring predators^17,28^. When black patterning is added to a typically aposematic colour, such as red, orange or yellow, it can sometimes enhance predator avoidance in the wild e.g.^29,30^, and under more controlled laboratory conditions can elicit unlearned aversions e.g.^8,31^, and increase the speed at which predators learn to avoid prey e.g.^32^. However, the benefits to prey of having high contrast patterning compared to being uniform aren’t always clearly evident^8,17,19,30,33,34^, and sometimes, naïve predators appear to be attracted to artificial prey with high luminance contrast as opposed to low contrast colour patterns^26,27^. This variation in results could depend on the stimuli being used or the foraging context^27,30^. In particular, the pattern manipulations could be inadvertently changing other aspects of prey appearance, such as the area of aposematic coloration or the overall mean luminance of the prey, which potentially confound the results. Therefore, despite studies exploring if patterning enhances predator avoidance, as yet there has been no critical test of the idea that the luminance contrast within aposematic patterns per se may influence their deterrent effect.

Here, we explicitly manipulate the degree of pattern contrast to test if high luminance contrast in aposematic signals is important for deterring a predator with excellent colour vision. We gave naïve domestic chicks repeated presentations of artificial prey where we could carefully control the degree of contrast in their achromatic naturalistic patterns whilst controlling for the potentially confounding effects of mean luminance. We predicted that if high luminance contrast was important, naïve predators should be less likely to approach and attack prey with high- compared to low-contrast patterning.

## Methodology

### Experimental subjects and housing

A total of 110 newly hatched chicks, of mixed sex, were bought from a commercial hatchery in two separate batches (55 chicks per batch) in two separate weeks. Each batch of chicks was housed in a floor pen measuring 1.4 m^2^ with wood chippings and a small mound of straw on the floor. The pen was located in a laboratory that was maintained at 25 °C using room heaters located next to the floor pen. The laboratory was lit using fluorescent lights (T5 840 tubes with no UV component), which were set on an automatic 12:12 hour light:dark cycle. These were however switched off manually during training and testing when only the arena lights were used (see below). Water was available *ad lib*, as were chick starter crumbs, except when periods of food deprivation were necessary prior to training and test trials (see below). All chicks were marked with non-toxic child friendly marker pens and weighed each day; the weights were recorded and monitored for chick welfare purposes. All chicks gained weight steadily throughout the experiment. At the end of the experiment all birds were humanely euthanized.

### Experimental arena

**Figure 1:**
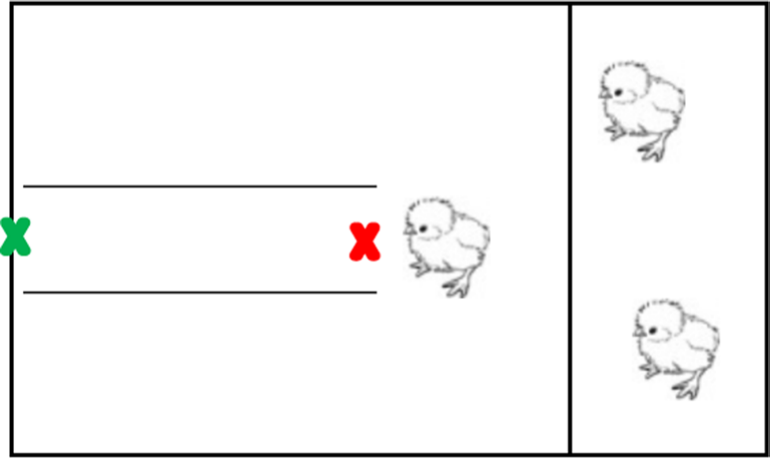
Diagram of experimental arena (seen from above), showing the ‘buddy area’ on the right-hand side, and the experimental area with the runway to the left. The distance between the start point (red cross), where the chicks were introduced to the runway, and the centre of the rear wall (green cross), where the prey were individually placed, was 50 cm. The walls of the runway were 15 cm apart and were at 90° to the rear wall. The runway ensured that chicks were only able to approach prey items pinned to the rear wall directly from front on, and not at an angle from the left or the right.

The training and test trials took place in an experimental arena which was located in the same laboratory as the floor pen. The arena was lit using four daylight bulbs (SoLux MR16 GU5.3, 4700 K, 35 W) during training and testing. It measured 100 × 50 cm, including a section measuring 25 × 50 cm fenced off with wire mesh to form a ‘buddy area’ (see Fig. 1). The buddy area contained food and water and housed two buddy chicks during the training and experimental trials. These were in view of any chicks in the experimental arena and prevented them from becoming stressed from isolation^35^. The buddy chicks were replaced at regular intervals from a pool of 14 individuals (7 in each batch). The floor of the arena was covered with plain white cage paper. A 50 cm long white plastic runway was placed in the arena. This was open at one end and terminated in a wall covered in white paper (100% recycled bright white 80 g/m^2^ paper, OfficeDepot, Boca Raton, FL, USA) (see Fig. 1). Consequently, it created a corridor along which chicks could walk to reach the artificial prey that we pinned to the end wall (see below). A camcorder was positioned above the experimental arena and recordings were made of the experimental trials to allow for detailed behavioural analyses afterwards.

### Artificial prey

To create moth-like prey, mealworms were pinned behind triangular shaped pieces of paper (isosceles: base 44 mm, height 37 mm), and placed on the back wall of the arena, at the end of the runway (see green cross in Fig. 1). In training, all of the paper triangles were white, but in the experimental test trials the triangles were printed with one of four pattern types. We used a 2 × 2 design, where the internal contrast of the pattern was either low or high and the pattern either contained only spots or included stripes as well. Contrast was defined as the Michelson contrast of the patterns, i.e. (R_max_ − R_min_)/(R_max_ + R_min_), where R_max_ and R_min_ are the maximum and minimum reflectance within a pattern, respectively. The two levels of Michelson contrast considered were 0.15 (for the low contrast patterns) and 1 (for the high contrast patterns). We selected the two types of patterns, containing spots and stripes, because they are features often associated with aposematic displays e.g.^18^.

**Figure 2:**
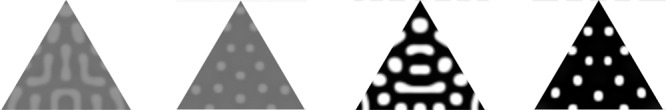
Examples of prey stimuli from each of the four experimental groups, from left to right: Low contrast stripes, Low contrast spots, High contrast stripes and High contrast spots.

The patterns were generated using a general reaction-diffusion model^36^. This is an example of a model that uses classic ‘Turing patterns’, i.e. mathematically defined patterns that emulate natural animal patterning^37,38^. To reflect the general diversity such models typically produce, and the diversity of aposematic patterns that are typically reported, we generated two main classes of patterns, one which had arrangements of spots, and another one which had striped areas (see Supplementary Methods for details). To obtain symmetrical patterns, as is the case for the left and right wing patterns of butterflies and moths, the corresponding images were then mirrored and cropped to form triangular targets. The targets were printed on 100% recycled bright white 80 g/m^2^ paper (OfficeDepot) at 600 dots per inch using a calibrated HP Color LaserJet Enterprise M552 printer (Hewlett Packard). The printer calibration was carried out using a hyperspectral imaging system consisting of an ultraviolet hyperspectral imaging camera (Resonon Pika NUV: Resonon Inc., MT USA) covering the 350–800 nm spectral range, with a spectral resolution of 1 nm, and the spectral sensitivity of chicken double cones receptors corrected for oil droplet absorbance^20^. Overall, the process resulted in four types of patterns, according to the level of contrast and whether the pattern contained only spots, or had striped areas (Low contrast-stripes, Low contrast-spots, High contrast-stripes and High contrast-spots; Fig. 2). Within each category of prey, we created 30 variants in an attempt to capture the natural variance that is found (both within and between species) amongst the wing patterns of Lepidoptera in nature^39^. In the experimental arena, the luminance of the darkest and lightest grey levels of the patterns were 13 and 216 cd/m^2^ respectively (measured with Minolta Luminance Meter LS-100; Konica Minolta Inc., Tokyo, Japan).

### Training trials

All experimental chicks were trained to forage alone in the experimental arena and to attack artificial prey pinned to the back wall of the runway. On Day 1, each chick spent six two-minute trials foraging for a mixture of chick crumbs and mealworms on the floor of the runway. This was to ensure that they became familiar with the arena and the runway. For the first two trials chicks foraged in groups of three, for the following two trials they foraged in pairs, and in the final two trials they foraged individually. Prior to the last three trials on Day 1, and all the following training trials, chicks were food-deprived for a maximum of 60 min to ensure that they were motivated to attack prey.

On Day 2, chicks were trained to eat mealworms pinned to the rear wall of the experimental runway. Each chick received four training trials at regular intervals throughout the day. In each trial, a single mealworm was pinned to the centre of the rear wall 10 cm from the runway floor. Chicks were placed at the entrance of the runway and left there until they attacked and ate the mealworm, or for 5 min, whichever came first. On Day 3, the chicks were again given four training trials at regular intervals throughout the day. However, in these trials, a white paper triangle was pinned over the mealworm so that it was only partly visible to the chicks. The base of the triangle was positioned in the centre of the back wall, 10 cm above the runway floor. On Day 4, each chick received a further four training trials, but now the paper triangles covered the mealworms completely. A chick had to approach and eat the mealworm within one minute in four consecutive trials on Day 4 to be included in the test trials. In total, 16 chicks did not reach the criterion and were removed from the study. This left a total of 80 chicks, with each chick randomly assigned to one of four experimental groups (Low contrast stripes: N = 19; Low contrast spots: N = 21; High contrast stripes: N = 20; High contrast spots: N = 20). The reason why subject numbers were not quite equal across groups was due to an error whereby one chick was mistakenly given prey with low contrast spots instead of low contrast stripes.

### Test trials

Across Days 5–6, each chick was given a total of five test trials following a food deprivation period of up to 60 min. In each trial, the chick was placed at the entrance to the runway, with a mealworm pinned to the rear wall, behind a triangular shaped piece of paper. Each triangle displayed a printed pattern, the visual properties of which differed according to experimental group (Low contrast stripes, Low contrast spots, High contrast stripes and High contrast spots; see above for details). Each chick was presented with a patterned triangle taken at random from the variants printed within their experimental group, and thus did not receive identical prey patterns across each test trial. A chick was removed from the arena once it had attacked and eaten the prey item, or after 3 min, whichever came first.

### Data collection and analysis

Adobe Premiere Pro (Adobe, San Jose, CA, USA) was used to analyse the videos of the experimental trials, with an image overlaying the videos that added 5 cm demarcations along the runway. Chicks were defined as approaching the prey if they were within an attacking distance of 15 cm from the prey. We also recorded whether or not a chick attacked (defined as touching the prey with their beak) and ate the prey. Since all of the mealworms that were attacked were subsequently eaten and there were no rejections post-attack, we just analysed if prey were eaten.

Our analyses focussed on the proportions of prey approached and eaten by all chicks as we were primarily interested on the selective benefits to prey. We used generalised linear mixed models (GLMMs) with binomial error and logit link function, with ‘approach’ or ‘eaten’ as the binary (0 or 1) response variable. We initially included trial number (1 to 5), pattern type (spots or stripes), luminance contrast level (low or high) and batch (first or second) as fixed factors in our full factorial models. Chick was always included as a random effect. Since batch was not a significant factor in either model (approach: F_1,379_ = 0.001, P = 0.982; eat: F_1,379_ = 0.125, P = 0.724), we pooled our data and removed batch from the subsequent analyses. There was no significant three-way interaction between trial, pattern type or contrast level in the full factorial model for the probability that prey were approached (F_1,379_ = 0.961, P = 0.429) and removing this interaction term significantly improved the fit of the model (AIC reduced from 2207.5 to 1992.7). We did not remove the non-significant three-way interaction from the model analysing the number of prey eaten since this did not improve the fit of the model (AIC slightly increased from 1928.7 to 1930.5).

For completeness, we also analysed the proportion of prey eaten of those approached to be able to directly compare decision-making at each stage. We used the same model, and once again, batch was not a significant factor (F_1,301_ = 0.479, P = 0.501) and was excluded from our main analysis. There was no significant three-way interaction between trial, pattern type or contrast level in the full factorial model (F_4,302_ = 0.909, P = 0.251) and removing this interaction term significantly improved the fit of the model (AIC reduced from 1.804.2 to 1617.9).

### Ethical statement

The study was approved by Newcastle University Animal Welfare and Ethical Review Body (Project ID No. 652), and performed in accordance with UK Home Office Guidelines and the Association for the Study of Animal Behaviour Guidelines for the Treatments of Animals in Research and Teaching.

## Results

### Prey approached

**Figure 3:**
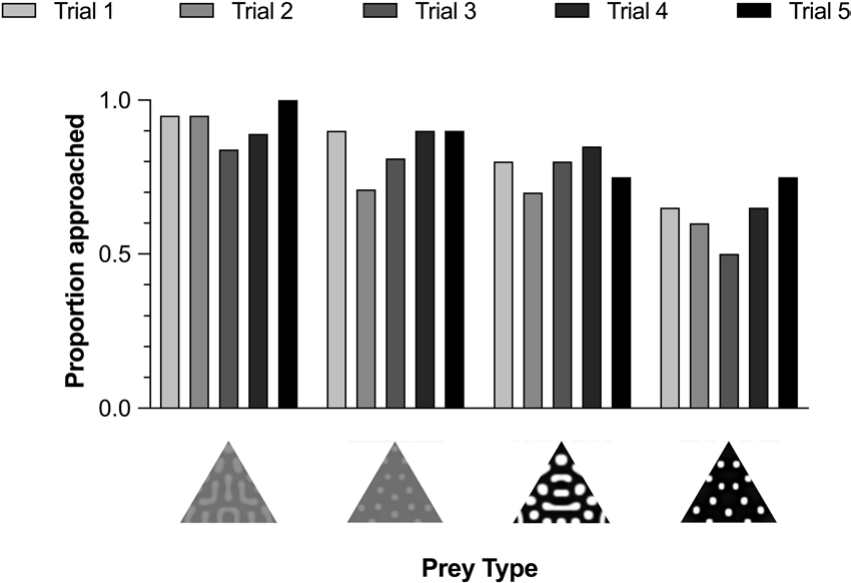
The proportion of each prey type that was approached across the five test trials by chicks in each of the four treatment groups, from left to right: Low contrast stripes, Low contrast spots, High contrast stripes, and High contrast spots.

More chicks approached prey with low contrast than high contrast patterns (F_1,384_ = 8.840, P = 0.003; Fig. 3). There was no effect of either pattern type (F_1,384_ = 2.369, P = 0.125), or trial (F_4,384_ = 1.689, P = 0.152). There were no significant two-way interactions (contrast x pattern type: F_1,384_ = 0.039, p = 0.844; contrast x trial: F_4,384_ = 0.425, p = 0.791; pattern type x trial: F_4,384_ = 0.360, p = 0.837). Therefore, these data suggest that luminance contrast was the main factor influencing chicks’ decisions to approach the patterned prey.

### Prey eaten

**Figure 4:**
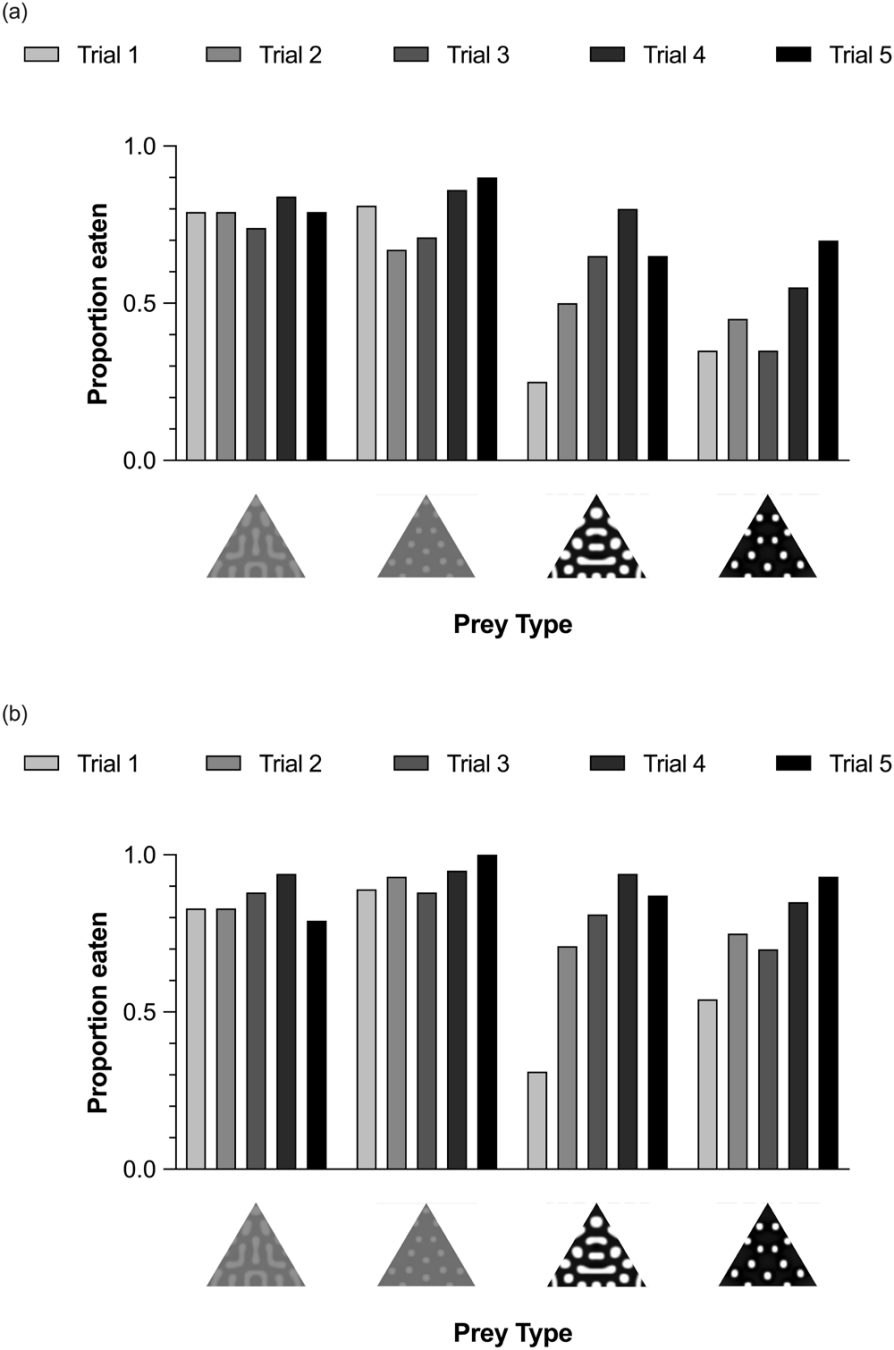
The proportion of each prey type eaten in each of the five test trials by chicks in the four treatment groups including data from: (**a**) all chicks, and (**b**) those chicks that approached the prey.

When chicks approached their prey, they didn’t always eat them. Using the data from all chicks, the prey with high contrast patterns were less likely to be eaten than those with low contrast patterns (F_1,380_ = 10.449, p = 0.001; Fig. 4a), irrespective of their pattern type (F_1,380_ = 0.408, p = 0.523). However, there was a significant effect of trial (F_4,380_ = 4.227, p = 0.002), as the proportion of prey that birds ate increased across trials. There was a tendency for this to be more pronounced in the groups given high contrast prey, although this interaction did not reach significance (contrast x trial: F_4,380_ = 2.167, p = 0.072). No other interaction was significant (contrast x pattern type: F_1,380_ = 0.383, p = 0.536; pattern type x trial: F_4,380_ = 0.965, p = 0.427; contrast x pattern type x trial: F_4,380_ = 0.904, P = 0.461).

When we only considered those prey that were approached, the results were qualitatively the same. Prey with high contrast patterns were less likely to be eaten than those with low contrast patterns (F_1,306_ = 2.905, p = 0.018; Fig. 4b), regardless of whether they had spots or stripes (F_1,306_ = 1.146, p = 0.285). Again, there was a significant effect of trial (F_4,380_ = 4.425, p = 0.002), since prey were more likely to be eaten in later trials. This seemed to be a stronger effect in the high contrast prey, although the interaction between level of contrast and trial did not reach significance (contrast x trial: F_4,306_ = 2.086, p = 0.083). No other interaction was significant (contrast x pattern type: F_1,306_ = 1.493, p = 0.223; pattern type x trial: F_4,306_ = 0.486, p = 0.746).

## Discussion

Our study clearly shows that increasing the luminance contrast in a prey’s warning pattern deters naïve predators: chicks were less likely to approach and eat prey with high contrast compared to low contrast patterns. This suggests that the high luminance contrast found in patterns of many aposematic prey, which often involve spots and stripes, could elicit unlearned biases and enhance survival even against predators with excellent colour vision, as well as those that have to rely on achromatic vision alone^28^. Whilst the likelihood of approach remained relatively stable across trials for all patterned prey, they were more likely to be eaten, and eaten once approached, with repeated encounters. Therefore, any survival benefits are predicted to wane if prey do not have an effective defence.

Given the costs and risks associated with eating harmful or toxic prey^5,35,40^, it is not surprising to find that predators have adapted to be cautious about approaching and eating unfamiliar prey^41,42^, particularly those that have visual features associated with aposematism, including being conspicuously coloured e.g.^15,43^, or having typical warning colours, such as yellow and red e.g.^8,11–13,44–47^. Whilst some studies have shown that pattern features can elicit stronger unlearned aversions in birds when they mimic the spatial arrangement of warning patterns e.g.^6,8^, our study is the first to show that the high luminance contrast between pattern elements, found in so many aposematic signals, can also elicit similar unlearned aversions. This is consistent with the empirical demonstration of reduced predation by wild birds on high contrast achromatic patterns that resemble eyes and the hypothesis that their aversive properties lie, at least in part, in their high internal contrast^48^. However, in these experiments, the prior experience of the predators was unknown. Therefore, whilst it has been suggested that high contrast patterns might be beneficial to aposematic prey faced with experienced predators, such as making them easier to recognise and remember^24^, we highlight how they can also enhance their survival against naïve predators.

Whilst the patterns with high luminance contrast were particularly effective at deterring naïve predators, even their survival benefits waned over repeated encounters. Interestingly, the likelihood that our prey were approached remained fairly constant over the five trials, and it was the likelihood that they were subsequently attacked and eaten that increased over time. This could be considered akin to ‘dietary conservatism’, where birds appear willing to approach novel prey but reluctant to attack them and include them in their diets^49,50^. Therefore, if dietary conservatism preceded the evolution of aposematism, patterns with high luminance contrast could have facilitated the initial evolution of warning signals through making naïve predators more wary of mutant prey and allowing warning signals to spread within a population^51–53^. The role of patterning has not been specifically considered in the context of dietary conservatism and the initial evolution of warning signals, but it could have been important in favouring a route to aposematism.

Although we found a clear effect of luminance contrast in the prey patterns on the birds’ decisions to approach and eat prey, the type of pattern, whether spotted or striped, did not seem to influence their behaviour. This may not be that surprising given that our pattern types were based on what we know about aposematic patterns, which often contain stripes and spots^18^. However, intriguingly, increasing luminance contrast in prey patterns doesn’t always need to be aversive to predators. High luminance contrast is important for disruptive camouflage, breaking up the body outline of prey and making them harder to find^54,55^. However, if prey have high contrast markings away from their edges, or the degree of contrast in pattern elements is greater than that of their backgrounds, prey become more likely to be detected and predated^54,56^. One possibility is that the effects on predators of increasing luminance contrast are pattern-dependent, and that predators are only wary of high contrast patterns if they contain elements commonly found in aposematic prey (e.g. spots, stripes or patterns that appear to highlight the body outline). It would, therefore, be interesting to determine how manipulating the level of luminance contrast within images of both cryptic and aposematic prey influences how predator respond to them.

Since we used achromatic stimuli in our experiment to focus solely on how luminance contrast could deter predators, we currently don’t know how luminance contrast and colouration might interact to enhance aversion, or which might be more effective at deterring predators. Studies show that changing the colours within a pattern e.g.^13,30^, or adding a black pattern to a base colour e.g.^8,57^ can alter the degree to which predators avoid prey. However, since coloration and internal contrast both co-vary in these manipulations, differences in avoidance behaviour could be due to changes in either, or possibly both^17,58^. It is also intriguing that the degree of difference in the attacks made on our high and low contrast prey (Fig. 4a) is comparable to that seen in studies comparing attacks on prey that have typical aposematic and non-aposematic colours e.g.^8,11,59^. However, although it’s tempting to conclude that luminance contrast may be as important as colour in eliciting unlearned aversions in birds, it is important to remember that these earlier studies used less controlled visual stimuli and very different methods. Testing the relative importance of colour and luminance contrast in unlearned aversions in birds is methodologically challenging, since it requires being able to match these two types of contrast quantitatively. This cannot be answered using current available models of avian vision, and has only been tackled in humans through a careful calibration of individual observers’ perception^60^. This is certainly an intriguing question for future research. However, our data highlight the need to consider the role of luminance contrast when comparing the effectiveness of different warning patterns in deterring predators in the laboratory or the field, and suggests that it may be more important in the function of aposematism than previously thought.

## Supplementary information

Supplementary information.

## Data Availability

The datasets generated during the study are available via the Newcastle University Data Repository: https://zenodo.org/record/3783735#.XrwXuBNKg6g].
