# Supplementary material for: Pattern contrast influences wariness in naïve predators towards aposematic patterns
Source: Sci Rep. 2020 Jun 8;10:9246. doi: 10.1038/s41598-020-65754-y (PMC7280217; doi:10.1038/s41598-020-65754-y)
Supplement: Supplementary file 1 — Supplementary information. [file 41598_2020_65754_MOESM1_ESM.pdf]

Pattern contrast influences wariness in naïve predators towards aposematic patterns

Halpin CG[1]*, Penacchio O[2]*, Lovell PG[3], Cuthill IC[4], Harris J[2], Skelhorn J[1], Rowe C[1^]

Supplementary Methods

Using Turing pattern modelling to develop stimuli

We wanted the patterns used in our artificial prey to emulate natural animal patterning. We used a reaction-diffusion model to create the patterns [1]. The equations of the reaction-diffusion system we considered are:

$$\frac{\partial u}{\partial t} = D\delta\nabla^2 u + \ \alpha u(1 - r_1 v^2) + v(1 \ - \ r_2 u)$$

$$\frac{\partial v}{\partial t} = \delta\nabla^2 v + \ \beta v\left(1 + \frac{\alpha r_1}{\beta}\ uv\right) + u(\gamma + \ r_2 v),$$

where u and v are concentrations of two chemicals that depend on spatial position and time and define the patterning, and $D, \delta, \alpha, \beta, r_1, r_2$ and γ are parameters that constraint the interplay between u and v. The model parameters in the reaction-diffusion equations were, for the spotted patterns, $(D, \delta, \alpha, \beta, r_1, r_2, \gamma) = (0.466, 0.0015, 0.699, -0.96, 2, 2, -0.699)$, and $(0.366, 0.0015, 0.949, -0.96, 4, 1, -0.949)$ for the striped patterns. Numerically, the patterns were generated on a square domain of size 100 x 100 using a discretisation and a finite difference Euler integration scheme with time step 0.01 and periodic boundary conditions to solve the reaction-diffusion parabolic partial differential equations [2]. The starting concentrations of the morphogens defining the initial conditions for the differential equations were drawn from uniform random distributions with values in the interval [0, 1]. The resulting patterns, which had values ranging between 0 ('black') and 1 ('white'), formed the pattern with high internal contrast (Michelson contrast 1). The low contrast patterns were obtained by reducing the range of reflectance values around 0.5 by scaling the high contrast patterns around this value in such a way to obtain a Michelson contrast of 0.15. The full process resulted in the creation of four types of patterns (see Figure 2 in main text). The patterns were next up-sampled to form 512 x 512 pixel images using Matlab's interpolating function 'griddata' with the 'cubic' method [3]. To obtain symmetrical patterns, as is the case for the left and right wing patterns of butterflies and moths, the corresponding images were next then mirrored to form images of size 512 x 1024 pixel. The triangular shaped pieces were then defined using isosceles triangles where the main height coincided with the axis of symmetry of the mirrored images (length, 512 pixels) and the base length was 610 pixels.

Chromatic contrast in the paper stimuli

The printed stimuli were designed to be greyscale patterns. However, as the printing device uses four colour primaries, it is fully possible that the printing process induces colour contrast between the two different values of grey in the patterns, at least for the patterns with a low contrast (the black area of the patterns with high contrast has a low photon catch and therefore does not trigger a chromatic response). To gauge the amount of chromatic contrast in the paper stimuli with low contrast, we computed the chromatic response to the two grey levels defining these patterns according to Osorio et al.'s model of colour vision of domestic chicks [4]. This model describes opponent channels defined by the interaction of the four types of cone receptors sensitive to (L) long-wavelength (M) medium-wavelength, (S) short-wavelength, or (U) ultraviolet light. Many opponent channels are possible in theory. We followed the common solution of modelling red-green (L-M) and yellow-blue ((L+M)-S) opponency, as in human colour vision, plus the avian-specific UV-blue (U-S) opponency. We assumed a CIE standard D65 daylight illuminant similar to the illuminant used to light the experimental arena (see Methods) and computed the photon catches of the four types of cones (L, M, S and U) and of the double cones. We found that the differences in chromatic signal between the two shades of grey in all opponent channels (formulas (4) for L-M opponency, (5) for (L+M)-S opponency and (6) for U-S opponency in the reference above) were low, as indicated by coefficient of variation (standard deviation divided by the mean value) in the table below (Table S1). By contrast, the coefficient of variation for the achromatic channel (double cones) was about 25%.

Table S1. Chromatic and achromatic contrast between the shades of grey defining the low contrast patterns.

	L-M	(L+M)-S	U-S	Double cones
Light grey	-0.0618	0.1857	-0.0581	3902.2
Dark grey	-0.0620	0.1771	-0.0613	2743.3
Contrast (in standard deviations)	0.0027	0.0333	0.0360	0.2498

References

1. Barrio, R. A., Varea, C., Aragon, J. L., Maini, P. K. A two-dimensional numerical study of spatial pattern formation in interacting Turing systems. *Bull. Math. Biol.* **61,** 483-505 (1999).

2. Strogatz S. H. *Nonlinear dynamics and chaos: with applications to physics, biology, chemistry, and engineering*. Boulder, CO:Westview Press (2015).

3. MATLAB *v. 9.3.0 (R2017b)*. Natick, Massachusetts: The MathWorks Inc (2017).

4. Osorio D., Vorobyev M., Jones C. D. Colour vision of domestic chicks. *J Exp Biol* **202,** 2951-2959 (1999).
